# Supervision of professional nursing practice in Brazil: a scoping review

**DOI:** 10.1590/0034-7167-2023-0077

**Published:** 2023-12-04

**Authors:** Andrezza Gabrielly dos Santos Soldera, Letícia da Silva Penha, Dieimes Leandro da Silva, Sebastião Junior Henrique Duarte, Rodrigo Guimarães dos Santos Almeida

**Affiliations:** I Universidade Federal de Mato Grosso do Sul. Campo Grande, Mato Grosso do Sul, Brazil; II Conselho Regional de Enfermagem de Mato Grosso do Sul. Campo Grande, Mato Grosso do Sul, Brazil

**Keywords:** Nursing, Nursing Audit, Professional Review Organizations, Review, Ethics Nursing, Enfermería, Fiscalización Sanitaria, Organizaciones de Normalización Profesional, Revisión, Ética en Enfermería, Enfermagem, Fiscalização Sanitária, Organizações de Normalização Profissional, Revisão, Ética em Enfermagem

## Abstract

**Objectives:**

to map studies that analyze the audit process of nursing councils.

**Methods:**

this is a scoping review, anchored in the JBI framework, with the guiding question: what is the evidence of the audit process of legal practice of nursing by class councils (COFEN/COREN system)? The searches were carried out in October and November 2022 without limitation of language and year.

**Results:**

of the 9 selected studies, all are Brazilian and published from 2014 onwards. Among the topics addressed are the role, challenges, costs and difficulties in nurse auditors’ daily work process, in addition to the contribution of the audit sector in Brazil.

**Conclusions:**

the studies gathered discuss aspects related to costs, challenges and difficulties, but there is no focus on corrective, disciplinary and educational activities as well as little is said about the audit process, its reporting, referral and outcomes.

## RESUMO

**Objetivos:**

mapear os estudos que analisam o processo de fiscalização dos conselhos de enfermagem.

**Métodos:**

trata-se de uma revisão de escopo, ancorada no referencial do JBI, com a questão norteadora: quais as evidências do processo de fiscalização do exercício legal da enfermagem pelos conselhos de classe (sistema COFEN/COREN)? As buscas foram realizadas em outubro e novembro de 2022, sem limitação de linguagem e ano.

**Resultados:**

dos 9 estudos selecionados, todos são brasileiros e publicados a partir de 2014. Entre os temas abordados, estão o papel do enfermeiro fiscal, desafios, custos e dificuldades no cotidiano do processo de trabalho, além da contribuição do setor de fiscalização no Brasil.

**Conclusões:**

os estudos reunidos discutem os aspectos relacionados a custos, desafios e dificuldades, mas não há enfoque nas atividades corretivas, disciplinares e educativas, bem como pouco se fala sobre o processo de fiscalização, suas notificações, encaminhamento e desfechos.

## RESUMEN

**Objetivos:**

mapear estudios que analizan el proceso de supervisión de los consejos de enfermería.

**Métodos:**

esta es una revisión del alcance, anclada en el marco del JBI, con la pregunta guía: ¿Cuál es la evidencia del proceso de inspección del ejercicio legal de la enfermería por parte de los consejos de clase (sistema COFEN/COREN)? Las búsquedas se realizaron en octubre y noviembre de 2022, sin limitaciones de idioma ni año.

**Resultados:**

de los 9 estudios seleccionados, todos son brasileños y publicados a partir de 2014. Entre los temas abordados están el papel del enfermero supervisor, desafíos, costos y dificultades en el proceso de trabajo diario, además de la contribución del sector de inspección en Brasil.

**Conclusiones:**

los estudios reunidos discuten aspectos relacionados a costos, desafíos y dificultades, pero no hay foco en las actividades correctivas, disciplinarias y educativas, así como poco se dice sobre el proceso de inspección, sus notificaciones, derivaciones y resultados.

## INTRODUCTION

In Brazil, Federal Law 5,905/1973 created Federal (COFEN) and Regional (COREN) Nursing Councils. These are public authorities that discipline nurses’, nursing technicians’ and assistants’ and midwives’ professional practice. The legal framework of nursing professions is also anchored in Federal Law 7.498/1986, Decree 94,406/1987, and among other regulations that aim to ensure the legal and ethical dictates of nursing^(1-2)^.

The law that created the nursing councils established that it is the responsibility of the federal government to regulate norms to be followed by the regional ones, aiming at the fulfillment of their final activities, with emphasis on the audit of professional practice. Audit procedures are contained in COFEN Resolution 617/2019, which standardized the guidelines and instruments at the national level^(3)^.

Among the objectives of monitoring the professional practice of nursing is to ensure qualified assistance to society as a whole by qualified people. This involves certifying the regularity of each worker by auditing an identity document and the individual registration database. Documents such as care protocols, internal regulations, certificate of technical responsibility, service schedules and other items that may compromise assistance to the population are also checked^(3-4)^.

Nurses who took a public exam or were appointed to the role are auditors, and federal and regional councilors also have the prerogative to supervise. The performance of auditors is based on educational, preventive, disciplinary and corrective approaches, seeking the quality of care provided to society and guaranteeing professionals’ rights to practice their profession, in addition to ensuring the implementation of the code of ethics and guaranteeing quality of care^(3-5)^.

Audit can be a planned action, or arising from annual planning, which takes into account the total number of health institutions registered with the Ministry of Health, but complaints are also attended to. In the latter situation, it is investigated whether the denounced fact has implications for professional practice and affects society. All audits are filed in an administrative process, consisting of the term that designated the action, proof of reports, letters and other documents that both identify irregularities and illegalities as well as responses^(3)^.

Even about 50 years after the creation of nursing councils, responsible for disciplining and auditing the professional practice of the contingent of almost 3 million workers, who are part of the largest workforce of the Brazilian Health System (SUS - *Sistema Único de Saúde*)^(6)^, even so, there are few literatures that can reveal the contributions of an ancient profession to society. The evidenced gap between legislation and practice highlights the difficulties encountered in the work process that interfere with the quality of care provided. In this regard, the following question was defined: what is the evidence of the audit process of the legal exercise of nursing by the class councils (COFEN/COREN system)?

## OBJECTIVES

To map the studies that analyze the audit process of nursing councils.

## METHODS

### Ethical aspects

As this is research with materials in the public domain that did not involve human beings, approval by a Research Ethics Committee was not necessary.

### Study design

This is a scoping review, which sought to map studies that analyzed the audit process of nursing councils. The study was developed according to the JBI Review Manual guidelines and structured through the following steps: 1) elaboration of guiding question and review objective; 2) search strategy elaboration; 3) search in databases; 4) selection of articles based on the reading of titles and abstracts; 5) selection of scientific articles based on their full reading; 6) summarization of results; and 7) presentation and discussion of results found^(7)^.

### Data sources and search strategies

To formulate the guiding question and search strategy, mnemonic PCC (Population, Concept and Context) was used. Population is the advice of professionals in nursing. The concept is the audit of professional practice by the nursing class council. Context is the legal practice of nursing professionals.

Articles that contained the three elements of PCC, without language or publication time limitations, were included. Articles that did not respond to the guiding research question, leaflets, or those whose full texts were not found online were excluded.

From the research question, controlled and indexed descriptors from Medical Subject Headings (MeSH), EMBASE Subject Headings (EMTREE), CINAHL Headings and Health Sciences Descriptors (DeCS) were selected. To combine these, Boolean operators OR and AND were used. Chart 1 presents the descriptors and keywords used for each mnemonic item.

**Chart 1:** Descriptors and keywords used in searches, 2023

Mnemonics	Descriptors	DeCS/MeSH/Emtree
**Population** Nursing councils	Professional Review Organizations	*Organizações de Normalização Profissional* *Círculos Médicos* *Comissão de Avaliação de Exercício Profissional* *Federações Médicas* *Normalização de Padrões Profissionais* *Organizações de Controle da Profissão* *Organizações de Controle de Padrões Profissionais* *Organizações de Revisão por Pares* Professional Review Organizations Review Organizations, Professional PRO Professional Review Organizations Organizations, Professional Review Organization, Professional Review Professional Review Organization Professional Standards Review Organizations PSRO Peer Review Organizations Organizations, Peer Review Organization, Peer Review Peer Review Organization Utilization and Quality Control Peer Review Organizations Professional standards review organization
**Concept** Audit of professional practice of nursing class councils	Sanitary Audit	*Fiscalização sanitária* *Fiscalização* *Fiscalização da Saúde* *Fiscalização em Saúde* *Monitoramento da Saúde* *Monitoramento em Saúde* *Monitoramento Sanitário* Nursing Audit Audit, Nursing Audits, Nursing Nursing Audits Nursing Audit
**Context** Legal practice of nursing	Ethics, Nursing	*Ética em enfermagem* *Ética de Enfermagem* Ethics, Nursing Ethic, Nursing Nursing Ethic Nursing Ethics Medical ethics

### Data collection and organization

The searches took place in October and November 2022, through remote access to the databases, from registration in the Coordination for the Improvement of Higher Education Personnel (CAPES - *Coordenação de Aperfeiçoamento de Pessoal de Nível Superior*) journal portal, via the Federated Academic Community (CAFe - *Comunidade Acadêmica Federada*), in the following databases: National Library of Medicine (PubMed/MEDLINE); Scopus; Embase; Web of Science; Scientific Electronic Library Online (SciELO); LILACS; and CINAHL with Full Text. Gray literature was also included in the sample, consisting of theses, dissertations and manuals related to the subject as well as those mentioned in the references of studies.

### Data analysis

The search for studies was carried out by two researchers independently and concurrently. In case of divergence, a third reviewer would be consulted, but there was no need. The selected studies were exported to the Mendeley software for organization and exclusion of duplicates. Therefore, the articles were migrated to the Rayyan application^(8)^ for initial screening with reading of titles, abstracts and subsequent assessment regarding the inclusion criteria.

To compose the final sample of results, the articles were retrieved in full, analyzed, and inserted into a spreadsheet prepared by the researchers in Microsoft Excel. For the purpose of analysis, they were numbered and coded and the results were presented in the form of charts and discursive report.

In order to ensure methodological rigor, the Preferred Reporting Items for Systematic reviews and Meta-Analyses extension for Scoping Reviews (PRISMA-ScR)^(9)^ checklist was used, in line with JBI recommendations. Furthermore, it was registered in the Open Science Framework (OSF) platform, under DOI: 10.17605/OSF.IO/FXHY8.

## RESULTS

**Figure 1:**
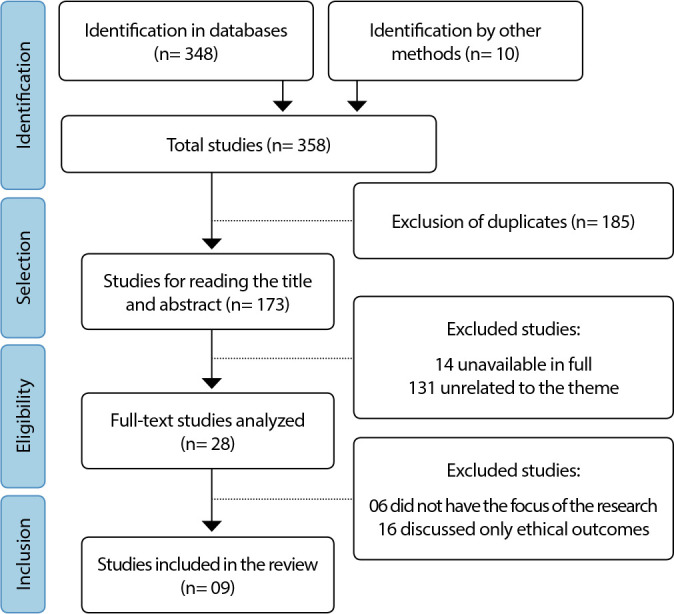
Study selection flowchart, 2023

Initially, 358 studies were identified in database searches and other sources. After the selection process, described in Figure 1, the final sample was constituted with 9 studies, 8 articles and 1 dissertation.

All included studies were written in Portuguese, and five were also available in English. All studies were conducted in Brazil and published in Brazilian journals. Following the CAPES Qualis classification for the four-year national nursing journals 2017-2020, four articles were published in B1, three in A2 and one in A4.

As for the year, the studies were published between 2014 and 2022, five of them after 2019. Although still scarce in the literature, there is an increase in relation to the theme over the years. Regarding authorship, two studies, related to the challenges and suffering of auditors, showed similarity between the authors, in addition to two other studies, as one discusses the audit process and the other about process costs, presenting the same authorship.

As for the study design, six studies were documental, three characterized as a case study and one cross-sectional, five with a qualitative approach and four quantitative. The studies were characterized and distributed, as shown in Chart 2.

**Chart 2:** Characteristics of studies included according to identification, objective, development region, approach, main results and contributions related to the audit process, 2023

ID^*^	Objective	Region	Approach	Mais results	Contributions
S1^(10)^	Analyze the audit process of professional practice in the COFEN/COREN system, identifying the difficulties related to its implementation.	-	COFEN/COREN system	There was a need to strengthen an audit process focused on the educational and participatory dimension, in accordance with COFEN Resolution 374/2011.	The strengthening of the audit process focused on COFEN Resolution 374/2011 principles contributes to patient care quality, nursing service regulation, in accordance with the law of professional practice and development of the health sector in Brazil.
S2^(11)^	Know the challenges and difficulties encountered in nurse auditors’ daily work process.	South region	COREN	Among the challenges cited by auditors are a lack of knowledge of regulation of professional practice, code of ethics and resolutions and decisions, and an outdated and/or negative view attributed to the council. Among the difficulties are issues related to the slowness resulting from the bureaucracy involved and the lack of standardization of the audit process.	It offered subsidies to improve nurse auditors’ work process, contributing to greater knowledge of all professionals about this work, since nurse auditors have the perception that they are an agent of change in this conception, strengthening professional work.
S3^(12)^	Identify the frequency and intensity of causes of moral distress experienced by nurses.	South, Southeast, North, Northeast Midwest regions	COREN	Participants revealed a higher frequency of causes of moral distress in two moments: first, associated with the slow audit process and insufficient human resources; second, associated with ethical problems, working conditions and difficulty ensuring quality of nursing care.	Reflecting on the causes of moral suffering is recognizing the need for an assessment of professional behavior and discussion on professional ethics, because changes must take place in the base that sustains the profession’s ethical precepts and moral conduct, in order to strengthen the category.
S4^(13)^	Assess the results of the ethical-professional audit of nursing in Nursing Homes.	Southeast region	COREN	A significant improvement was verified between the first and the last audit in relation to the legality of professional practice. Issues such as irregular registration in the council, classification of care without a standard and professionals from other classes on the nursing scale showed greater resolution, however, regarding the dimensioning of nursing, there was no difference.	The observed results are sensitive to audits when they translate parameters of advances in the quality of services offered to institutionalized residents and also in comprehensive nursing care.
S5^(14)^	Identify the characteristics of the audit of professional practice carried out by four professional councils in health.	-	COFEN	All professions in health analyzed have a law creating their class councils. In nursing, medicine and pharmacy resolutions regulate the audit system operation; however, in dentistry, it was not identified. Nursing was the only class that showed characteristics of an audit process with educational precepts, encouraging ethical values and valuing the work process in its regulation.	The importance of making national norms available by the councils is highlighted, in order to ensure the uniformity of audit and guarantee patient safety from the professions’ legal and regular exercise. Moreover, health audit standardization is important for the development of professions with an interdisciplinary focus.
S6^(15)^	Discuss the results-oriented governance and strategic management process of the COFEN and COREN system final and administrative activities.	-	COFEN/ COREN system	CORENs’ audit sector is part of the Division of Audit of Professional Exercise (DFEP) of COFEN. COFEN works by carrying out technical visits, training and monitoring the conduct adopted by CORENs.	Audit activities correspond to 2.27% of the autarchy’s total budget, an amount destined to seek uniformity and strengthen the audit work process.
S7^(16)^	Analyze audit acts in public institutions in Rio de Janeiro related to neonatal care.	Southeast region	COREN	The precariousness of audited health institutions and the lack of material and human resources were identified during document analysis, in addition to verification of the course time of the process, which was considerably long.	The process of audited institutions made it possible to observe the delay in progress, the lack of closure of processes and the growing process of judicialization, reflecting the audit sector’s and COREN’s legal department in that state.
S8^(17)^	Identify the average direct cost related to the direct labor of the auditors involved in the “on-site audit” of the audit process carried out at the COREN-SP Headquarters Unit.	Southeast region	COREN	The average cost of the initial “on-site audit” is 1.29 times greater than that of the return “on-site audit”, corresponding to R$ 331.67 (US$66.33) and R$ 256.16 (US$51.23) on average, respectively. Time (p ≤ 0.001) and cost of the initial “on-site audit” (p ≤ 0.001) are greater than for the return “on-site audit”.	Contributions related to costs can be justified by the fact that, in order to perform the initial “on-site audit”, the auditor needs to request information and assess the questions contained in the terms used, in addition to auditing the sectors where there is a nursing service, since, in the return audit, it is verified that the inadequacies reported in the initial audit have been remedied.
S9^(18)^	Describe the “on-site audit” sub-process of the “audit” macro-process, carried out at the COREN-SP Headquarters Unit.	Southeast region	COREN	The description of the initial and return “on-site audit” subprocess facilitated the identification of activities that add value and relevance to the audit process.	The activities and tasks pre-established in the audit script, inserted in the respective audit terms, need to be carried out in a detailed and sequential manner, aiming at compliance with current legislation.

* *ID - study identification.*

Among the selected studies, researches that involved auditor nurses’ role, challenges and difficulties in the daily work process as well as the costs related to auditors’ direct labor stand out.^(11-12,18)^. Other studies have highlighted the importance and characteristics of COFEN’s and COREN’s audit sector in Brazil and their contribution to improving quality of care^(10,13-17)^.

## DISCUSSION

The audit of professional practice in nursing occurs through a continuous, dynamic process permeated by several actions, guided by COFEN Resolution 617/2019^(3)^. This is a subject of great relevance for the advancement of the profession, but there is a lack of studies on the subject, making the subject of audit something veiled by professionals and managers.

Research available in the literature focuses on discussions based on ethical-disciplinary processes, which can be characterized as one of the developments of audit, but it is important to emphasize that this development is not the only one, considering the multiple referrals resulting from audit. The situation may be related to professionals, who failed to comply with some determination of the council, conditions of professional irregularity, or to the institution, such as in situations of opening a public civil action or ethical interdiction^(16)^.

Of the studies included in the review, one of the repercussions addressed is related to the lack of conformity of reports by institutions and professionals, evidenced by research that sought to analyze audit acts, in addition to the main challenges, difficulties and moral suffering encountered in nurse auditors’ daily work^(11 12,16)^. Auditors, when verifying the management documents, protocols, duty rosters, records and nursing notes, staff sizing, implementation of the Systematization of Nursing Care (SNC) as well as all the factors that involve the work process, aim to ensure safe nursing care within legality, in addition to preventing failures. However, a study that analyzed the legal outcomes of errors in perioperative care and in delivery and birth care related to nursing identified lawsuits for errors related to the absence of nursing records and execution of procedures that are not the responsibility of the nursing team^(3,19)^.

The peculiarities related to the audit activity in the COFEN/COREN system were also addressed by one of the studies included in the sample that sought to discuss the governance and strategic management process in relation to the COFEN/COREN system’s final and administrative activities. CORENs’ audit activities are linked to DFEP, technical body responsible for executing the necessary strategies for the execution of management guidelines and policies in the area of audit of professional practice, with the objective of standardizing and consolidating the actions that involve professional practice audit^(15)^.

In relation to professional advice in the health sector, research observed that, when analyzing the professions of nursing, dentistry, medicine and pharmacy, nursing is the only one that has an audit process based on Resolutions with an educational character, encouraging ethical values and valuing the work process^(14)^. However, the need to consolidate these precepts and strengthen these activities is discussed, in order to expand legislation into practice^(10)^.

The reductionism of the activities carried out and the lack of knowledge of the work carried out by the councils, on the part of some professionals, whether they are managers, managers or assistants, may be related to their social value. Research carried out with nurses and nursing technicians on how they saw the activities of nursing councils identified in subjects’ speeches that the audit activity was reported in a punitive perspective^(20)^. In order to combat this linked view, COFEN Resolution that conducts the councils’ auditory activity guides that actions must be guided within an educational and participatory dimension, based on pedagogical and open actions, focusing on the quality of care and not merely punitive^(10)^.

During audits, the auditors focus on complying with legislation related to professional practice regulation^(19)^. Nursing team activities involve clinical practice activities and technical responsibility, which highlights the inseparability of care and management dimension of the nursing work process. However, in professional practice, it is perceived that there is a difficulty in articulating the dimensions, in which professionals who perform managerial activities tend to value this action in order to support the viability of care; on the other hand, those who are in care tend not to value the management activity, attributing it a purely bureaucratic nature^(21)^.

Several challenges are related to the audit process of professional practice of nursing. In research carried out with a view to nurse auditors’ work activities, divergences with the institutions visited and a lack of ethical-legal knowledge among nurses were identified. However, a difficulty highlighted by the auditors is related to the lack of standardization of the audit process^(11-12)^. In order to minimize this problem, COFEN, through COFEN Resolution 617/2019, brings new strategies for operating the professional exercise audit system, considering that CORENs can request changes according to each reality so that they do not violate the which is advocated^(3)^.

In the international context, a process similar to enforcement is carried out through regulatory laws. However, not all countries and states have the same legal regime, and the divergences found between the places suggest the international political weakening of the profession, in addition to opening up room for greater tolerance of ethical infractions, especially since, in some countries, more than an agency carries out this regulatory process^(22)^.

Nurse auditors’ work, as it involves complex activities permeated by administrative and hierarchical procedures, can be fragmented and often time-consuming, if they do not follow the standardized determinations by COFEN. Two studies carried out with nurses auditors from CORENs’ audit departments mentioned a lack of some resources, the main ones being human and technological^(11-12)^.

The lack of human resources can become a limiting factor, since the need to prioritize the institutions to be audited due to their insufficiency was cited^(12)^. However, in relation to technological resources, investments should serve as subsidies to increase efficiency in order to consider the balance between audit costs and available financial resources^(23)^.

In relation to costs and time spent on audit activities, variables were analyzed in a study carried out in COREN’s audit department in the state of São Paulo, with an average of R$ 299.15 (US$59.83) and 171 minutes in the first audit of the institution. There are also some elements that can help to increase the time, such as institution size, complexity of services offered and auditors’ specific knowledge and prior experience in the area^(17)^. This information contributes to the system, by generating subsidies to increase the efficiency and effectiveness of the human resources required in the audit process, since audit corresponds to 2.27% of the federal agency’s total budget^(15)^.

The various facets and issues involving the spheres of regulation, audit and professional performance are important for the functioning of nursing services. Audit educates the people involved and combats actions that disrespect the laws, in favor of public interest, individual rights and freedoms, contributing to the improvement of the quality of services provided to the population.

### Study limitations

Difficulties related to lack of a specific descriptor limited the searches, since most articles used “Health Regulation and Audit” as a descriptor; however, the definition does not converge with the subject studied, being considered an essential function in public health, whose objective is “to develop and/or improve regulatory frameworks and laws as well as the execution of activities to ensure compliance with regulations in a timely manner, congruent and complete”. The unavailability of some articles in full can be considered a limitation found in the data collection procedure. Moreover, the scarcity of international articles that discuss the audit of the professional practice of nursing in health services limits the discussion only to Brazil.

### Contributions to nursing, health, or public policies

Understanding the importance of the activity to guarantee social rights, quality in services and nursing care, in addition to recognizing nursing class councils’ work can support advances in the area. Openness and encouragement for research such as the present one represent a fundamental initiative and commitment to the audit process qualification.

## CONCLUSIONS

COREN and COFEN play an important role in regulating nurses’, technicians’ and nursing assistants’ practice, and even with the 27 states of the federation composing the COFEN/COREN system and complying with audit, there is still a lack of dissemination of actions performed, which limits the contributions of the largest professional contingent in the health area.

The audit of professional practice of nursing in Brazil is still little explored, which justifies the need for further studies in the area. The process is discussed, aspects related to costs, challenges and difficulties are discussed, but there is no focus on corrective, disciplinary and educational activities as well as little is said about reports, referrals and outcomes.

Through the set of guidelines that involve the audit process, assistance by a qualified professional is guaranteed, organizing the work process in nursing and safeguarding the society’s interests of public health and well-being.
